# Amphibian skin bacteria display antifungal activity and induce plant defense mechanisms against *Botrytis cinerea*

**DOI:** 10.3389/fpls.2024.1392637

**Published:** 2024-04-09

**Authors:** Yordan J. Romero-Contreras, Francisco Gonzalez-Serrano, Damien Formey, Wendy Aragón, Florencia Isabel Chacón, Martha Torres, Miguel Ángel Cevallos, Julian Rafael Dib, Eria A. Rebollar, Mario Serrano

**Affiliations:** ^1^ Centro de Ciencias Genómicas, Universidad Nacional Autónoma de México, Cuernavaca, Morelos, Mexico; ^2^ Programa de Doctorado en Ciencias Biomédicas, Centro de Ciencias Genómicas, Universidad Nacional Autónoma de México, Cuernavaca, Mexico; ^3^ Instituto de Biociencias, Universidad Autónoma de Chiapas, Tapachula, Chiapas, Mexico; ^4^ Planta Piloto de Procesos Industriales Microbiológicos (PROIM) - Consejo Nacional de Investigaciones Científicas y Técnicas (CONICET), Tucumán, Argentina; ^5^ Instituto de Microbiología, Universidad Nacional de Tucumán, Tucumán, Argentina

**Keywords:** frog skin microbiota, biological control, Botrytis cinerea, Arabidopsis thaliana, blueberries

## Abstract

*Botrytis cinerea* is the causal agent of gray mold, which affects a wide variety of plant species. Chemical agents have been used to prevent the disease caused by this pathogenic fungus. However, their toxicity and reduced efficacy have encouraged the development of new biological control alternatives. Recent studies have shown that bacteria isolated from amphibian skin display antifungal activity against plant pathogens. However, the mechanisms by which these bacteria act to reduce the effects of *B. cinerea* are still unclear. From a diverse collection of amphibian skin bacteria, three proved effective in inhibiting the development of *B. cinerea* under *in vitro* conditions. Additionally, the individual application of each bacterium on the model plant *Arabidopsis thaliana, Solanum lycopersicum* and post-harvest blueberries significantly reduced the disease caused by *B. cinerea*. To understand the effect of bacteria on the host plant, we analyzed the transcriptomic profile of *A. thaliana* in the presence of the bacterium C32I and the fungus *B. cinerea*, revealing transcriptional regulation of defense-related hormonal pathways. Our study shows that bacteria from the amphibian skin can counteract the activity of *B. cinerea* by regulating the plant transcriptional responses.

## Introduction

*Botrytis cinerea* is a necrotrophic fungal pathogen that causes gray mold disease, a devastating infection affecting a wide variety of economically important crops such as tomato, cucumber, pepper and strawberry (Dik & Wubben, 2007; Petrasch et al., 2019). Due to its broad distribution and ability to infect more than 250 plant species, it is considered the second most important agricultural pathogen (Haidar et al., 2016; Hua et al., 2018). This pathogen is characterized by its broad infectivity, causing damage to various tissues such as leaves, stems, flowers and fruits, particularly during the harvest, post-harvest and storage periods, resulting in significant losses in terms of quality and quantity of product (Roca-Couso et al., 2021; Bi et al., 2023). For decades, synthetic fungicides have been used to combat this pathogen, but their use has been restricted due to the adverse effects on the environment and human health and fungicide resistance acquired by several *B. cinerea* strains (Abbey et al., 2019; Fedele et al., 2020). In this context, there has been a growing interest in the development of new, more sustainable, ecological and environmentally safe alternatives for the control of this phytopathogenic fungus (Aladdin et al., 2018; Chacón et al., 2022).

Plants have developed several molecular strategies to counteract the effect of bacterial and fungal pathogens. Pathogen-Associated Molecular Patterns [PAMPs], such as chitin, are recognized by Plant Pattern-Recognition Receptors [PPRs], which activate plant immune signaling as a first line of defense. However, when the fungus crosses this barrier, the plant can counteract the effect of the pathogen through the activation of secondary responses, which involve the activation of hormones such as salicylic acid [SA], jasmonic acid [JA] and ethylene [ET], acting as key regulators of the defense against pathogens, such as *B. cinerea* (Mengiste et al., 2010; AbuQamar et al., 2017; El-Saadony et al., 2022). Finally, the induction of defense activation known as Systemic Acquired Resistance [SAR] and Induced Systemic Resistance [ISR] occurs (Choudhary et al., 2007). Despite these plant defense responses, *B. cinerea* can still infect a large number of crops, pointing out the suggesting the importance of identifying new ecofrendly alternatives. Plant disease biological control agents or BCAs refers to microorganisms, such as bacteria, fungi, and viruses [and/or their products], that suppress infections caused by a pathogen, through antagonism or induction of disease resistance, such as SAR and ISR (Babbal et al., 2017; O’Brien, 2017; Díaz et al., 2020; Pereyra et al., 2020). Besides preventing plant diseases, many BCAs also can promote plant growth, improve tolerance to abiotic stress and enhance nutrient acquisition (Babbal et al., 2017; El-Saadony et al., 2022).

Currently, it has been reported that many bacterial species can activate protective systems against plant pathogenic fungi (Aladdin et al., 2018). For example, *Bacillus* spp (Balthazar et al., 2022; Chacón et al., 2022), *Streptomyces* spp (Boukaew et al., 2017), *Pseudomonas* spp (Trotel-Aziz et al., 2008), *Acinetobacter* spp. (Zhang et al., 2019) and other bacteria are BCAs that help combat infections by *B. cinerea*. However, although some of these microorganisms are already used in the field, some concerns arise about their use, particularly due to the possible development of resistance to them by the pathogenic fungi (Di Francesco et al., 2018; Carmona-Hernandez et al., 2019). Moreover, there is still lack of information on the effect of BCAs on plant metabolism at the transcriptomic level (Ferreira-Saab et al., 2018). Recently, it was discovered that the use of amphibian skin bacteria have the potential to inhibit *Colletotrichum orbiculare*, the fungus causal of cucumber anthracnose (Susilawati et al., 2021). Additionally, we have recently described that bacteria isolated from neotropical frogs, belonging to the genus *Acinetobacter*, were able to inhibit the growth and development of *B. cinerea* (Cevallos et al., 2022).

In this work we investigate the mechanism used by three bacteria [C23F, C26G, and C32I], isolated from the frog *Craugastor fitzinger* to inhibit growth and development of *B. cinerea* and their involvement in the induction of the plant defense systems (Rebollar et al., 2016; Cevallos et al., 2022 *)*. We also determined that exogenous application of these bacteria and their filtrates protects the plants *Arabidopsis thaliana*, *Solanum lycopersicum* and blueberry fruits [*Vaccinium corymbosum*] against *B. cinerea* infection. Additionally, to understand the effect of the bacteria [*Acinetobacter* sp. C32I] we studied the transcriptional changes induced in *A. thaliana* and observed modifications in the expression levels of genes involved in plant defense. Based on these findings, we propose that the bacteria from amphibian skin have the potential to serve as promising biological control agents against plant pathogens.

## Materials and methods

### Antifungal activity assay for the selected bacteria against *B. cinerea*

Bacteria were previously identified by Rebollar et al. (2019). The bacteria C26G and C32I were recently described as new species of the *Acinetobacter* genus (Cevallos et al., 2022), while C23F was identified at the family level as *Enterobacteraceae* through 16S rRNA sequencing (Rebollar et al., 2018). All bacteria isolates were initially screened for their antifungal activity against the pathogen *B. cinerea* by the direct confrontation assay. For this, each bacteria was cultured on Luria-Bertani medium [LB] at 30°C for 24 h until an optical density of 0.6 [O.D_600nm_]. The spore suspension of *B. cinerea* strain B05.10 [provided by Brigitte Mauch-Mani] was prepared as previously described (L’haridon et al., 2011). Dishes with potato dextrose agar [PDA, Sigma-Aldrich], were inoculated with 10 µl of each bacterial suspension at one end of the plate and 6 µl of the spore suspension of *B. cinerea* at the other end and incubated in the dark at 24°C for 7 days. The test was done in triplicate [n=3]. The fungal growth inhibition was evaluated by measuring the area of mycelium growth with ImageJ2 software [version 2].

### Effect of bacterial filtrates on *B. cinerea* growth

Bacteria were cultivated in LB liquid medium, at 30 °C in the dark until an optical density of 0.6 [O.D_600nm_]. Each bacterial liquid culture was centrifuged at 12,500 rpm for 15 min and the supernatant was filtered through a 0.22 μm Millipore membrane. For the inhibitory assay, Petri dishes [60 mm] containing PDA were supplemented with 50, 60, 70 and 80% [v/v] of bacterial filtrates [BFs]. As a control, dishes with PDA were supplemented with LB medium. A 5 mm sterile filter paper disk with 6 μl of suspension of *B. cinerea* [5 × 10^4^ spores ml^−1^] spores was placed at the center of the dish and incubated at 24°C for 7 days in the dark. The test was done in triplicate [n=3]. Inhibition was evaluated by measuring the diameter of the mycelium on the dish. Images of growing *B. cinerea* mycelium were analyzed using the ImageJ2 analysis software [version 2].

### Effect of bacteria filtrates on the germination of *B. cinerea* spores

Three drops of a spore suspension of the fungus [5×10^4^ spores ml^−1^], including each bacteria filtrate at 80%, were placed on slides in a growth chamber at 24°C with >95% relative humidity for 24 h. Subsequently, the germinated spores were quantified in a Neubauer chamber. Staining of spores was performed (Romero-Contreras et al., 2019), and were observed under fluorescence microscope [Zeiss Axioskop 2, 40x].

### Effect of bacterial volatile organic compounds [VOCs] over *B. cinerea* growth

To detect the inhibitory activity of bacterial volatile organic compounds [VOCs], 20 μl of a cell suspension of C23F, C26G, and C32I bacteria [0.6 O.D_600nm_] was inoculated in the center of LB plates. Simultaneously, a suspension of *B. cinerea* spores [5×10^4^ spores ml^-1^) was inoculated on PDA medium plates. Subsequently, the bottom of each plate [fungus and bacteria] were placed face to face and immediately sealed with parafilm. The plates were then incubated at 26°C for 7 days, together with a control, which consisted of PDA plates inoculated with the pathogen and uninoculated LB plates. The experiment was conducted in duplicate [n=2]. Inhibition was assessed by measuring the diameter of the mycelium on the plates. The growth halo of *B. cinerea* was measured using the ImageJ2 software [version 2].

### Pathogen infection assays on *Arabidopsis thaliana*

Seeds of the ecotype Columbia-0 [Col-0, Nottingham Arabidopsis Stock Centre, Nottingham, UK] as wild type and the mutants of *eds5-1* (Nawrath and Métraux, 1999), *jar1* (Chassot et al., 2007) and *ein3* (Roman et al., 1995), were germinated on 0.2X Murashige-Skoog [MS] medium supplemented with sucrose [0.5% w/v], and 1% [w/v] agar (Murashige and Skoog, 1962) for 7 days. The plants were then transplanted into germination trays containing a mixture of soil and vermiculite [3:1] and kept in a greenhouse at 22 ± 2°C and 60% humidity with 16 h/8 h light/dark cycles for one month with constant irrigation of non-sterile water. Then, 1 ml of each bacterial cell suspensions or bacterial filtrates was added [adjusted to 0.6 O.D_600_] every five days for four weeks. For the pathogenicity assay, leaves of water-treated control or treated *A. thaliana* were infected with 6 µL of a suspension of *B. cinerea* spores [5×10^4^ spores ml^−1^]. The inoculated plants were covered with plastic lids to maintain >95% relative humidity and transferred to a growth chamber at 22 ± 2°C and a 24 h dark cycle. After 72 hours post-infection [hpi], symptoms were evaluated. To determine the percentage of plant survival, we quantified the number of infected plants [disease incidence]. The lesion sizes were quantified using the ImageJ2 software [version 2]. The experiment was performed with a randomized design with 45 plants [three leaves per plant] per treatment.

### RNA extraction and transcriptomic analysis of *A. thaliana leaves*

*Arabidopsis thaliana* Col-0 plants were root-inoculated with *Acinetobacter* sp. *C32I* and foliar infected with the *B. cinerea* B05.10. After 6 hours post-infection [hpi], ten leaves per plant were collected and preserved in liquid nitrogen. Conditions were set for both dual and tripartite interactions of the plant with each microorganism. Total RNA extraction was performed from two independent experiments using Trizol, following the supplier instructions [Invitrogen]. RNA concentration and purity were measured with a NanoDrop spectrophotometer [Implen NP80, Thermo Fisher Scientific, USA]. Afterwards, 1 μg of total RNA was treated with DNAse [Thermo Scientific™, USA] to remove the genomic DNA, according to manufacturer of instructions. Library construction and sequencing were carried out by the Beijing Genomics Institute [BGI] Americas 2 using DNBSeq TM technology. The sequences are publicly available in the NCBI with number project PRJNA986187 and PRJNA1044848. To identify differentially expressed genes [DEGs], DESeq2 software in the Integrated Differential Expression Analysis MultiEXperiment [IDEAMEX] was used, with a FoldChange ≥ 2 or ≤ -2, and an adjusted p-value ≤ 0.05 (Jiménez-Jacinto et al., 2019). The DEG analysis was grouped through Gene Ontology [GO] analysis using PANTHER [v17.0]. GO term enrichment for the plant analysis was performed using the TAIR web tool [The Arabidopsis Information Resource [TAIR], https://www.arabidopsis.org/tools/go_term_enrichment.jsp, on www.arabidopsis.org, Feb 24, 2022], analyzed with the Fisher test and FDR correction. Non-redundant enriched terms were obtained by using REVIGO software (Supek et al., 2011). The graphs were created using the ggplot2 library with RStudio and heatmap libraries [v4.2.1].

### qRT-PCR analysis

To analyze the expression of genes, a third independent biological replica was performed. Leaves from root-inoculated plants with *Acinetobacter* sp. C32I and foliar infected with the *B. cinerea* B05.10 were collected. RNA isolation using TRI Reagent^®^ [Sigma-Aldrich, United States], were performed following the instructions of the manufacturer. RNA integrity was evaluated by using denaturing gel electrophoresis and measured using NanoDrop spectrophotometer [Implen NP80, Thermo Fisher Scientific, USA]. Afterwards, 1 μg of total RNA was treated with DNase I [Thermo Fisher Scientific, Inc., Waltham, MA, USA]. For cDNA synthesis, we used 1 μg of the total RNA, and then processed with a RevertAid H Minus RevertAid First Strand cDNA Synthesis kit [Thermo Fisher Scientific, Inc., Waltham, MA, USA], according to manufacturer’s instructions. Primers for the RT-qPCR gene expression analysis were: *AOS* [S_5’-ATCCAAAGATCTCCCGATCC-3’, AS_5’-GTGGATTCTCGGCGATAAAA-3’], *ACS6* [S_5’-TGGTTGGTTAAAGGCCAAAG-3’, AS_5’-TGGTCCATATTCGCAAAACA-3’]*, PR1* [S_5’-TTCTTCCCTCGAAAGCTCAA-3’, AS_5’-AAGGCCCACCAGAGTGTATG-3’] *and ZAT12* [S_5’-ATCAAGTCGACGGTGGATGT-3’, AS_5’-ACAAAGCGTCGTTGTTAGGC-3’]. To normalized transcript abundance two housekeeping genes were used. *ACTIN* [S_5’-TGCTTTGCCACATGCTATCC-3’, AS_5’-GACTTCAGGGCATCGGAAAC-3’] and *CF150* [S_5’-CCGACAAGGAGAAGCTTAACAAGTT-3’, AS_5’-CGGCAGATTTGGATGGACCAGCAAG-3’]. The conditions of the reactions were performed according to Aragón et al. (2021).

### *In vivo* biocontrol assay with blueberry fruits

For *in vivo* infection assays, blueberries at commercial maturity were harvested from organic fields that had not received any preharvest treatment with synthetic pesticides. Subsequently, the fruits were disinfected with a 2% NaClO solution for 2 minutes, followed by washing with tap water, and then left to dry at room temperature. Bacterial cultures [C23F, C26G, and C32I] were grown to a density of 0.6 [O.D_600nm_]; subsequently, centrifuged at 12,500 rpm for 15 min. The supernatants were recovered and transferred through a 0.22 μm Millipore filter to obtain the bacterial filtrates [BF], which were then resuspended in physiological solution [NaCl 9 g/L], to achieve a concentration of 80% [v/v]. The cell pellets were dissolved in 3 ml of physiological solution. Blueberries were punctured to induce a lesion and were then independently sprayed with 3 ml of the bacterial solution and the BF. Subsequently, they were incubated at 25 ± 1°C and 95% relative humidity for 24 h. Three independent biological replicates, each one with 10 blueberries, were used for each treatment. Physiological solution served as the control. Following bacterial inoculation, blueberries were sprayed with a solution of *B. cinerea* ISIB-MMA/F-Bc01-S spores [1x10^6^ conidia/mL] and kept at 25 ± 1°C for 7 dpi. Infection was assessed by counting healthy and infected fruits (Chacón et al., 2022).

### Bacterial inoculations of *Solanum lycopersicum* plants

*Solanum lycopersicum* L. seeds were washed with a 3% solution of sodium hypochlorite [3 times] and absolute ethanol [1 time]. The seeds were germinated in hydrated vermiculite. After 7 days of growth, they were transferred to pots with a mixture of soil and vermiculite [3:1] and maintained in a greenhouse at 22 ± 2°C with 60% relative humidity under a 16 h/8 h light/dark cycle. Over five weeks, inoculations of the soil every three days were done with C32F, C26G, and C32I bacteria, grown in 0.2x MS liquid medium. Leaves were collected from plants pretreated with each of the bacteria and then infected with *B. cinerea* spores [5×10^4^ spores ml^-1^]. The plants were maintained under >95% relative humidity at 24°C for three days in a plant growth chamber with a 16 h/8 h light/dark cycle. Lesion evaluation was conducted with ImageJ2 software [version 2]. Three biological replicates were performed, each with 10 leaves per treatment.

### Statistical analysis

The data obtained from each test are presented in the figures as mean values [ ± SEM]. Statistical analyses were conducted using a One-way ANOVA followed by Tukey *post-hoc* test [p < 0.05] to identify significant differences for each experiment. The graphs were generated using R [https://posit.co/]. These analyses were based on results from three independent experiments.

## Results

### Antifungal activity of amphibian skin bacteria against *B. cinerea*

**Figure 1:**
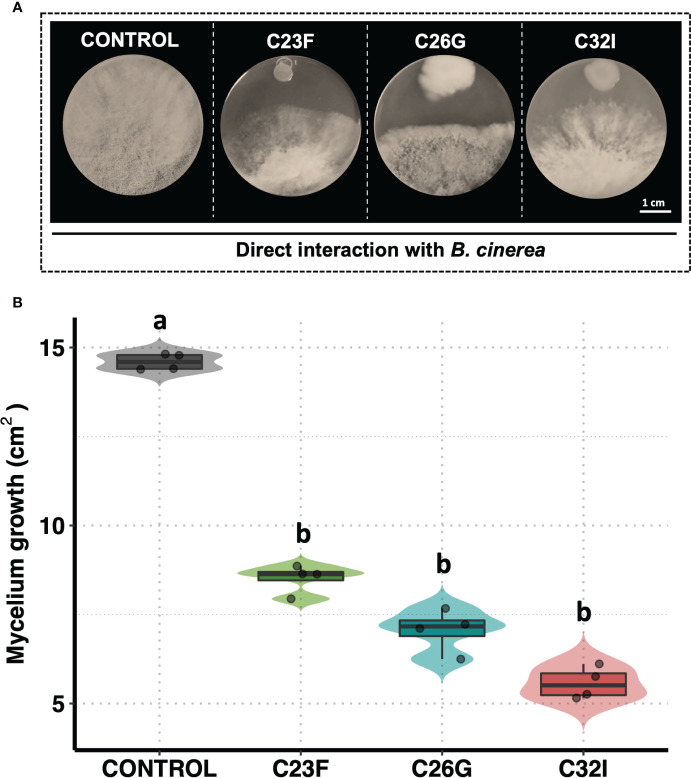
The C23F, C26G, and C32I bacterial inhibit the *B cinerea* growth in direct confrontation. The bacterial were inoculated in PDA medium with *B. cinerea* spore suspension [5×10^4^ spores ml^−1^] and incubated at 24°C for 72 (h) **(A)** Representative pictures of the inhibitory assay are included as a visual illustration. **(B)** The growth was evaluated by measuring the diameter of the mycelium on the dish compared with the control. Data represent the mean [ ± SD] of three independent experiments, each performed in duplicate, and presented relative to control. Letters indicate a statistically significant difference, according to one-way analysis of variance [ANOVA] [*p ≤ 0.05*] followed the Tukey test.

Previously, we had demonstrated the inhibitory activity of bacteria isolated from amphibian skin against *B. cinerea* (Cevallos et al., 2022). Based on these results, two more promising strains belonging to the genus *Acinetobacter* C26G and C32I were selected, and the reproducibility and consistency of their antagonistic behavior was corroborated. In addition, a third bacterium, C23F, previously classified at the *Enterobacteraceae* family level, was included (Rebollar et al., 2018). The results revealed that all three bacteria exerted an antagonistic effect on the growth of *B. cinerea* (**Figure 1A**). In particular, the measurement of mycelial growth of the fungus showed that the three bacteria were able to significantly inhibit the growth of *B. cinerea*, compared to the uninoculated control, where the fungus invaded the entire plate (**Figure 1B**). These results indicated that bacteria from frog skin inhibited *B. cinerea* growth under *in vitro* assays.

### The compounds released by frog skin bacteria inhibited the growth of *B. cinerea*

**Figure 2:**
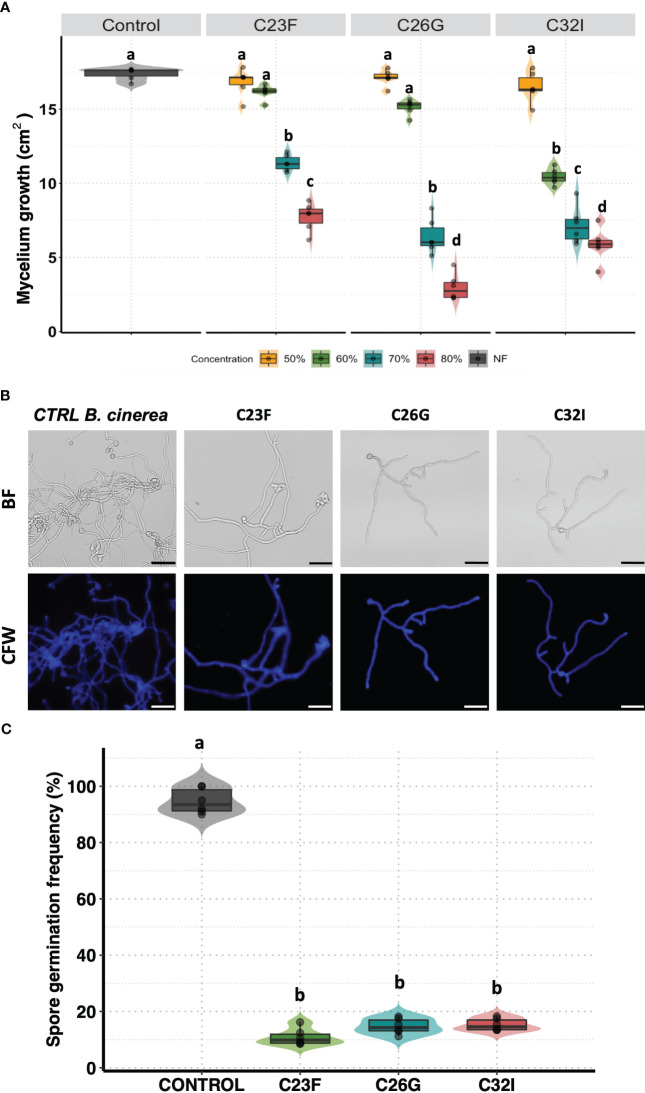
The filtrates of frog skin microbiota inhibit the growth *B cinerea*. Spore suspension of *B cinerea* [5 × 10^4^ spores ml^−1^] was placed on the center of the Petri dish containing PDA supplemented with different concentrations of BFs and incubated at 24°C to 72 (h) **(A)** The inhibition was measured calculated the area growth of *B cinerea* represented in percentage. The germination was observed co-cultured the *B. cinerea* spores with PDA supplemented with 80% BFs in the dark at 24°C for 24 (h) **(B)** The pictures represented the effect BFs in the germination de *B. cinerea.* Staining with Calcofluor-White [CFW], shows the integrity of the hyphae. **(C)** The development of the fungus was calculated by counting the number of spores germinated. Bars represent mean values [± SD] of three independent experiments. Letters indicate a statistically significant difference, according to a one-way analysis of variance [ANOVA] [*p* ≤ 0.05] following to the Tukey test. Scale bars, 20 µm.

Several BCAs have been shown to have the ability to secrete various compounds with antifungal activity that contribute to counteract the effect of pathogenic fungi (Ferreira-Saab et al., 2018). To investigate whether C23F, C26G, and C32I bacteria secrete compounds that interfere in the germination and growth process of *B. cinerea*, we confronted the bacterial filtrates [BF] with the pathogen. Our results revealed that the BF of each bacteria inhibited the growth of *B. cinerea* in a dose-dependent manner (**Figure 2A**). We observed that when the pathogen was grown on PDA medium supplemented with 50% and 60% BF, approximately 10% inhibition occurred for the three bacterial strains. However, at concentrations of 70 and 80% BF, a direct correlation in the inhibition of *B. cinerea* growth was observed (**Figure 2A**). To determine the effect of BFs on spore germination, we co-cultured a suspension of *B. cinerea* spores, along with 80% of BFs for 24 h (**Figure 2B**). The data showed that strain C23F influenced the germination rate of spores of 8.5%, followed by C26G and C32I with 11.33% and 13.76%, respectively, compared to the unfiltered control, where 100% germination was observed (**Figure 2C**). Our results indicate that BFs significantly inhibits the development and growth of *B. cinerea*, which highlights their potential use as BCAs.

### VOCs produced by bacteria can inhibit *B. cinerea* growth

**Figure 3:**
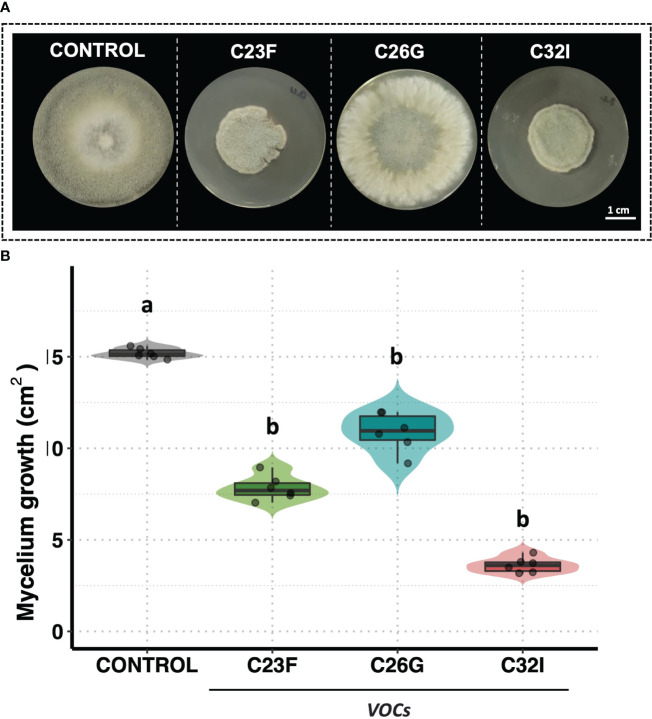
VOCs from bacteria C23F and C32I are involved in the inhibition of *B cinerea*. Double-sided experiments were performed, in which bacteria and fungi were grown in their respective culture medium. **(A)** Representative images of the inhibitory effect of the VOCs on the growth fungus mycelium. **(B)** The growth was evaluated by measuring the diameter of the mycelium on the dish compared with the control. Data represent the mean [ ± SD] of three independent experiments, each performed in duplicate, and presented relative to control. Letters indicate a statistically significant difference, according to one-way analysis of variance [ANOVA] [*p ≤ 0.05*] followed the Tukey test.

To test the antagonistic activity of volatile organic compounds [VOCs] in inhibiting *B. cinerea*, a double-sided assay was performed. The data revealed an inhibitory effect of the VOCs produced by two frog-associated bacteria [C23F and C32I] on the growth of the pathogenic fungus, in contrast to the control without bacteria, where mycelial growth was observed throughout the entire surface of the plate (**Figure 3A**). A quantification of the growth area demonstrated that the bacteria C26G had the least inhibitory effect against *B. cinerea*, exhibiting similar growth as the untreated control and covering a total area of approximately 15 cm². In contrast, VOCs from bacteria C23F and C32I displayed inhibitory effects on the fungus, resulting in growth areas of 6.58 cm² and 3.17 cm², respectively (**Figure 3B**). Interestingly, our findings revealed that, in addition to the release of diffusible organic compounds, volatile organic compounds are also produced, which might play a role in restraining *B. cinerea*.

### Frog skin microbiota contributed to the defense system in the plant against *B. cinerea*

**Figure 4:**
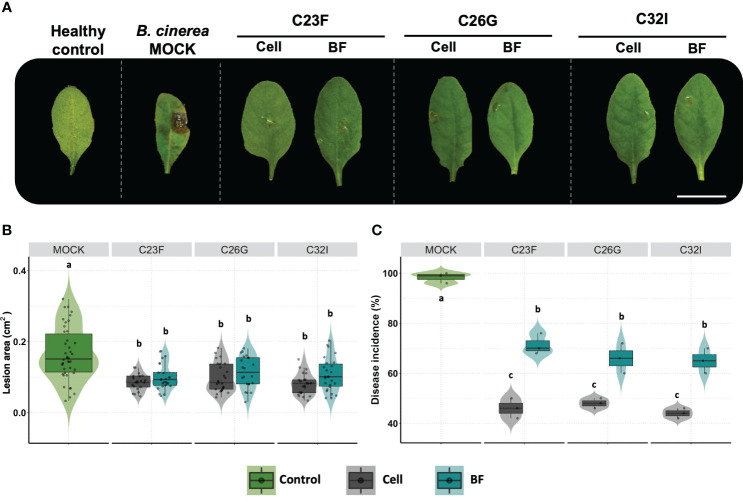
The cells and bacterial filtrates induced plant defense in presence of *B. cinerea*. Plants were inoculated with cells from the C23F, C26G and C32I strains, as well as their corresponding filtrates, and infected with pathogen. **(A)** The images show the impact of bacterial cells and filtrates on symptom development caused by *B. cinerea* after 3 days post-infection [dpi]. **(B)** Evaluation of lesion size in plants treated with bacteria. **(C)** Measuring of disease incidence in treated plants. The graphs represent two experiments were done [n=50, ± SD]. The letter indicate significance different, according to one-way analysis of variance [ANOVA] [*p ≤ 0.05*] followed by Tukey test. Mock represent the plants with MS medium.

Several biocontrol agents induce plant defense systems, which confer a protection system against pathogenic fungi (Wang et al., 2021). To analyze the effect of bacteria C23F, C26 and C32I directly on the plant, we inoculated the soil, in which *A. thaliana* plants were growing, with a suspension of cells or BFs and evaluated their effect against *B. cinerea* (**Figure 4**). Upon infecting *A. thaliana* leaves with *B. cinerea*, we observed that the lesions caused by the pathogen on the plants pre-treated with the bacterial cells or BFs were significantly smaller compared to the non-inoculated control after three days post-infection [3 dpi], (**Figure 4A, B**). Next, we measured the incidence of infection on the leaves and observed that plants inoculated with C23F cells and filtrates showed a disease incidence of 46% and 71% respectively, and plants treated with C26G showed 48% and 66% incidence, followed of plant inoculated with C32I with 44% and 65%, disease incidence, compared with the controls that showed a 98% incidence (**Figure 4C**). These results indicate that both the bacteria and the BF induce defense responses in the plant when present in the soil, protecting leaves against *B. cinerea* infection.

### Biocontrol activity of bacteria in blueberries against *B. cinerea* infection

**Figure 5:**
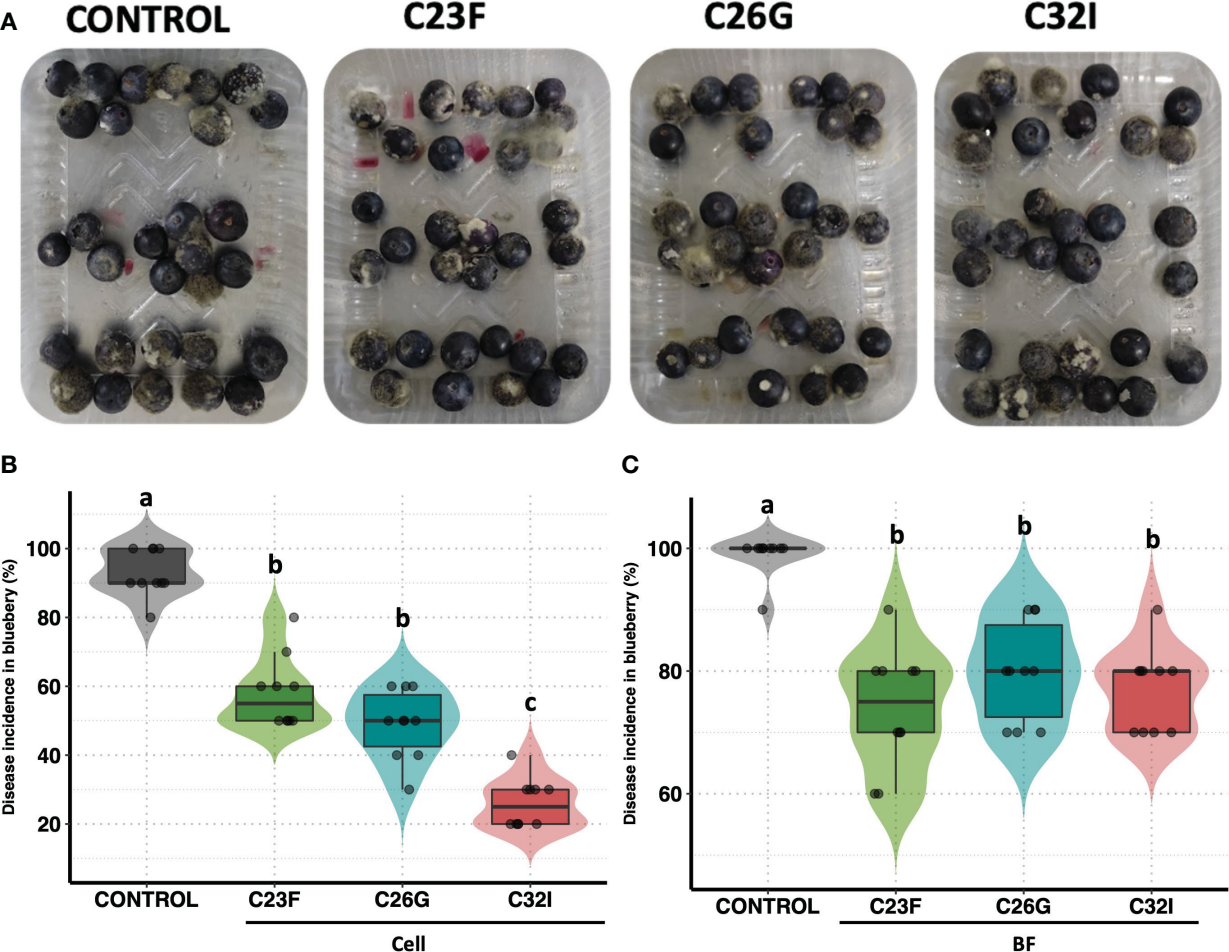
Biocontrol effect of bacterial cell and filtrates against *B. cinerea* on blueberries. Blueberries were treated with bacterial cells and filtrates [BF], and then infected with the *B. cinerea*. After 24 hours [hpi], photos were taken to evaluate the incidence. **(A)** Representative images of each treatment. **(B)** The graph represents the percentage incidence of the treatments with bacterial cells and filters. The analysis represents three independent experiments, each one with 10 blueberries. The letter indicate significance different, according to one-way analysis of variance [ANOVA] [*p ≤ 0.05*] followed by Tukey test. Control represents the fruits with physiological solution.

To analyze the effect of bacteria to protect commercial crops such as blueberries during post-harvest, we performed an *in vivo* test using either cells or BFs of C23F, C26G and C32I, and inoculating with *B. cinerea* ISIB-MMA/F-Bc01-S (Chacón et al., 2022). We observed that blueberries fruits, treated with bacteria and infected with the fungus, showed a lower lesion grade compared to the non-inoculated control (**Figure 5A**). In untreated condition, 95% of the fruits developed the disease, while the fruits treated with cells bacteria, showed incidences of 58%, 49%, and 26% for bacteria C23F, C26G, and C32I, respectively (**Figure 5B**). For the treatment of blueberries inoculated with BF, the pathogen incidence was similar to the treatment with bacterial cells. The control treatment showed an incidence of 99%, while the BF treatments showed incidences of less than 80% (**Figure 5C**). Additionally, we analyzed the protective effects of each bacterium against *B. cinerea* using leaves from tomato plants inoculated with the respective bacterium. Our results showed a reduction in lesions in plants treated with each bacterium, compared to the control group where lesions were significantly higher (**Supplementary Figure 1**). Overall, the results indicate that C23F, C26G, and C32I and their filtrates [BF] protect plants and fruits against *B. cinerea*, suggesting a potential for biological control applications.

### Transcriptional changes of *A. thaliana* in response to *Acinetobacter* sp. C32I and the pathogen *B. cinerea*

**Figure 6:**
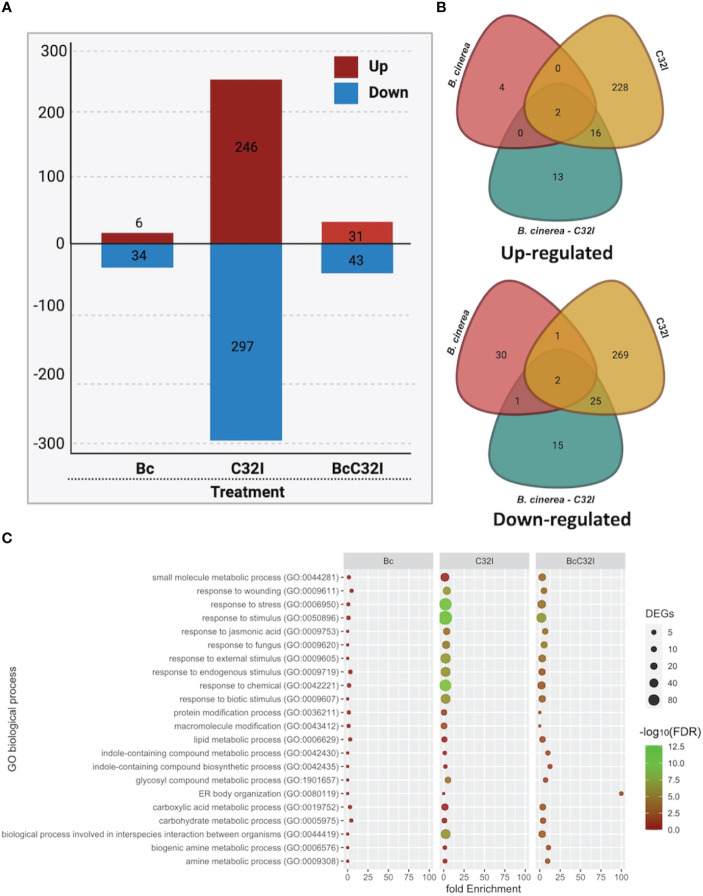
Transcriptome analysis of *A. thaliana* pathosystem with *Acinetobacter* sp. C32I and *B. cinerea* [6 hpi]. **(A)** Number of differentially expressed genes [DEGs] identified in the treatments, where red indicates up-regulated, and blue indicates down-regulated genes. **(B)** Venn diagrams show the number of up-regulated and down-regulated differentially expressed genes [DEGs] identified in the treatment compared to the control. **(C)** Distribution of the different biological functions of Gene Ontology [GO] for the up-regulated genes [p<0.05]. The graph represents the enrich “Biological process” GO-terms [FDR <0.01]. The color of the circles indicates the significance of the term [-log_10_ FDR], and the size of the dots represents the number of genes associated with that term. For the analysis of tripartite DEGs, we employed the Bc *vs* Bc-C32I comparison [Log2FC ≥ 2 or ≤ -2].

To investigate the transcriptional changes in the plant upon inoculation with the C32I bacterium and the pathogen *B. cinerea*, we conducted both bipartite and tripartite interactions among the organisms under greenhouse conditions. We observed a difference in the number of up-regulated and down-regulated genes (**Figure 6**; **Supplementary Data 1**). We identified that 6,246 and 31 DEGs up-regulated, while 34,297, and 43 DEGs were down-regulated for each treatments, respectively (**Figure 6A**; **Supplementary Data 2**). Additionally, we explored whether these DEGs were shared among the treatments: we did not find shared DEGs for all the treatments and only few were shared when two treatments were compared (**Figure 6B**).

To assess the potential functions of the identified DEGs, these genes were assigned to significant annotations by Gene Ontology [GO] term enrichment analysis and were classified with respect to biological processes (**Supplementary Data 3**). All DEGs were classified into 22 biological processes, including the biological processes involved in interspecies interaction between organisms, response to biotic stimulus, response jasmonic acid, response to fungus, among others (**Figure 6C**). The results suggests that plants in the presence of C32I and *B. cinerea*, triggers the expression of various functional groups that differ with respect to dual interaction.

### Genes related to plant defense responses are induced during the pathosystem in *Acinetobacter* sp. C32I and *B. cinerea*

**Figure 7:**
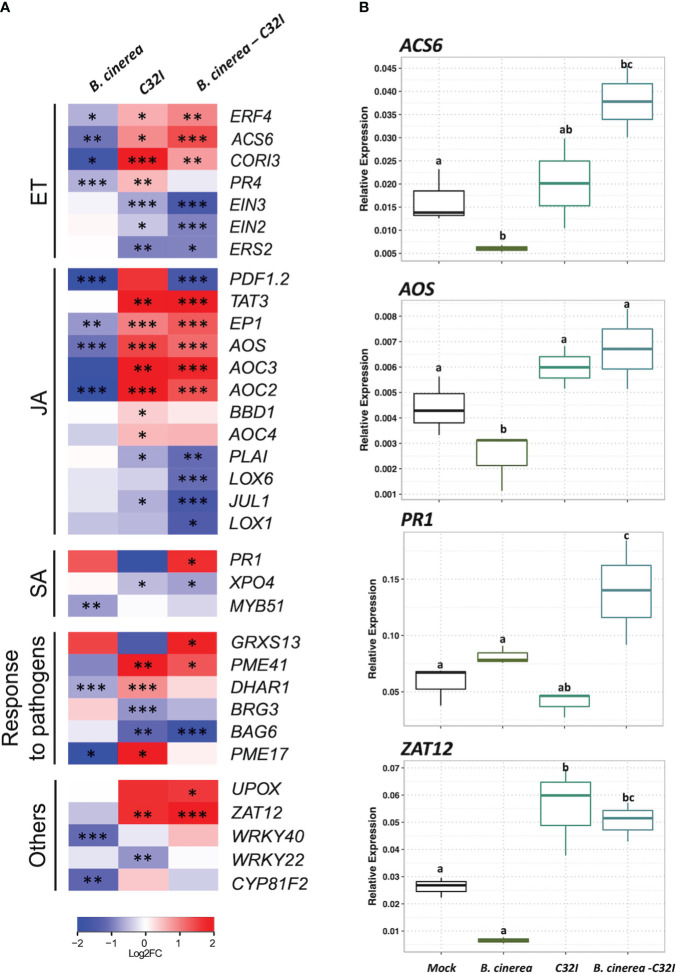
Expression analysis of jasmonic acid [JA]-, ethylene [ET]-, salicylic acid [SA]-response to pathogen and other genes in response to *B. cinerea* [6 hpi]. **(A)** Heatmap shows the expression levels of up-regulated genes. The colors represent the level of expression calculated between the control condition compared with each microorganism individually [dual interaction], and the tripartite interaction, ranging from blue [–2] to yellow [2]. **(B)** Expression analysis of genes *ACS6, AOS, PR1* and *ZAT12*, through RT-qPCR, based on Ct values, normalized with the expression of the two housekeeping genes. The data represent the means of three technical replicates. Error bars indicate ± SD. *p ≤ 0.05, **p ≤ 0.01, ***p ≤ 0.001.

To comprehend changes in the expression levels of genes associated with hormonal pathways, we performed a clustering of genes categorized into salicylic acid [SA]-, jasmonic acid [JA]-, ethylene [ET]-response to pathogen and others related to transcription factors. We analyzed the accumulation of transcripts in *A. thaliana* during the dual and tripartite interactions between the *Acinetobacter* sp. C32I and *B. cinerea* [6 hpi] (**Figure 7**). In the initial analysis, we compared the control condition [non-inoculated] with the plant exposed only to *B. cinerea*. In the ethylene [ET] pathway, we detected a down-regulation of genes, that participate in the resistance to *B. cinerea*. However, in the dual plant-bacteria interaction, we observed an induction of *ERF4, ACS6, CORI3* and *PR4* ET-related genes and down-regulation of the receptors *EIN2* and *EIN3* (Wójcikowska and Gaj, 2017; Jiang et al., 2023; Shin et al., 2023; Tiwari et al., 2023). Regarding tripartite interaction, the genes showed the same up-regulation, except for *PR4* gene. Concerning jasmonic acid [JA] pathway, we observed a down-regulation of the responsive genes in presence of fungus. On the other hand, in the interaction of the plant with C32I, is observed up-regulation of the *PDF1.2, TAT3, EP1, AOS, AOC3, AOC2, BBD1*, and *OC4* genes (Stenzel et al., 2003; You et al., 2010; Omidvar et al., 2021; Mathan et al., 2022), and these expression levels were maintained in the tripartite interaction. Furthermore, in the salicylic acid [SA] pathway, *PR1* gene was induced during the infection with fungus and during the tripartite interaction.

Additionally, we analyzed a group of genes that have been described to respond to the defense against pathogens. Notably, during plant-bacteria interaction condition, there was an up-regulation of the genes *DHAR1* [DEHYDROASCORBATE REDUCTASE] and *PME17* and *PME41* [PECTIN METHYLESTERASE], the latter recently identified in defense against *B. cinerea* (Del Corpo et al., 2020; Aragón et al., 2021). The expression levels were similar during the tripartite interaction to genes, including *UPOX* [UPREGULATED BY OXIDATIVE*]* and *ZAT12* [ZINC FINGER OF ARABIDOPSIS] that participate in oxidative stress (Davletova et al., 2005; Vaahtera et al., 2014). Taken together, our results demonstrate that in the presence of the C32I bacterium, the transcriptional response of the plant is modified, indicating an early activation of the plant defense system, that is maintained in during the tripartite interaction, suggesting that the bacteria induce the plant immunity against *B. cinerea*. Finally, we confirmed the expression patterns found with RNA-seq with the expression of four genes selected from each hormone pathway: *ACS6*, *AOS*, *PR1*, and *ZAT12* through qRT-PCR (**Figure 7B**).

### Resistance of *B. cinerea* is dependent on JA/ET

**Figure 8:**
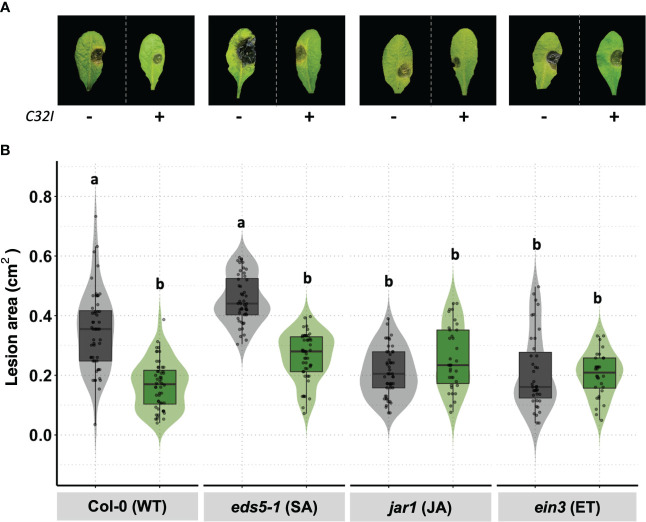
*Acinetobacter* sp. *C32I* treatment induces JA/ET-dependent resistance in *A. thaliana*. **(A)** Appearance of foliar symptoms in inoculated and non-inoculated plants. Representative leaves per genotype are shown. **(B)** Disease incidence of *B. cinerea* in wild-type *A. thaliana* [Col-0], and *eds5-1* [SA], *jar1* [jar1], and *ein3* [ET] mutants were tested. Experiments were repeated twice with similar results. The graphs represent the average of both experiments [n=50, ± SD]. Symptoms were determined at 3 days post-infection [dpi]. Letters indicate significant differences, according to one-way analysis of variance [ANOVA] [*p ≤ 0.05*] followed by Tukey test. Control represents plants with MS medium.

To elucidate the signal transduction pathways involved in the defense response induced by *Acinetobacter* sp. C32I against *B. cinerea*, the wild-type plant and mutants involved in the JA [*jar1*], ET [*ein3*], and SA [*eds5-1*] signaling pathways were inoculated with the bacterium C32I and infected with *B. cinerea*. The data demonstrated variation in the lesion size for each inoculated and non-inoculated treatment was detected (**Figure 8A**). An analysis of the lesion area in the Col-0 [WT] and *eds5-1* [SA] mutant showed a reduction in the lesion area caused by *B. cinerea* at 3dpi occurred when the plant was inoculated with the bacteria. However, the defense response in the *jar1* [JA] and *ein3* [ET] mutants showed the same lesion area, in both conditions (**Figure 8B**). Overall, the results suggested resistance of C32I-inoculated *A. thaliana* against *B. cinerea*, dependent on JA and ET signaling, rather than SA signaling.

### The *Acinetobacter* sp. C32I produces transcriptional changes different from other biocontrol agents

**Figure 9:**
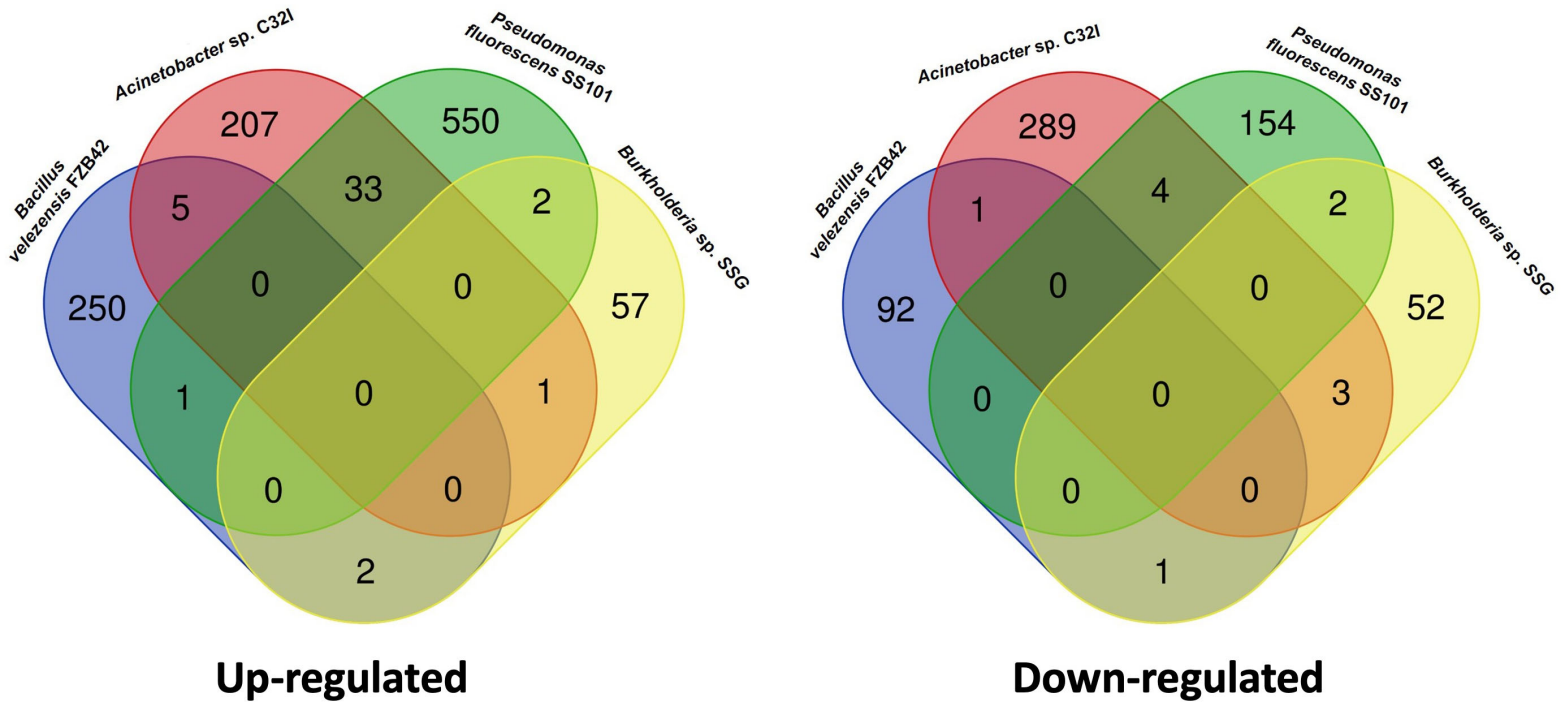
Comparative transcriptomic analysis with other biocontrol agents. Transcriptomic data of the bipartite interaction between *A. thaliana* with *Acinetobacter* sp. C32I, were compared with other biocontrols reported. Venn diagrams show the overlap of **(A)** up-regulated and **(B)** down-regulated genes.

We have observed that bacterium C32I induces transcriptional changes in the plant, manifesting differentially expressed genes [DEGs], both up-regulated and down-regulated, and some of the are related to defense systems. When comparing our results with other transcriptional reports of previously characterized biocontrol agents, we noticed a difference in the expressed genes in the presence of C32I, compared to *Bacillus valezensis FBZ42*, *Pseudomonas fluorescences SS01*, and *Burkholderia* sp. *SSG* (**Figure 9**) (van de Mortel et al., 2012; Tzipilevich et al., 2021; Kong et al., 2022). When we compared the transcriptional changes induced by C32I only few genes are shared with the other BCAs, suggesting that, even though all the analyzed BCAs have a biocontrol activity, transcriptional regulation by C32I triggers defense induction mechanisms distinct from other microorganisms.

## Discussion

In nature, plants and animals interact with a plethora of microorganisms (Compant et al., 2010; Khare et al., 2018). Some bacteria can establish a beneficial association with plants, favoring plant growth, enhanced defense responses, and tolerance against biotic and abiotic stresses (Jones et al., 2019; Kang et al., 2019). Similarly, amphibians are known to possess a microbial community in their skin, which protects them against multiple factors, including the necrotrophic fungus *Batrachochytrium dendrobatidis* (Rebollar et al., 2018; Varga et al., 2019; Rebollar et al., 2020). It has been described that the amphibian skin and the phylosphere of the surrounding plants can share microbiomes, that can influence the tolerance to biotic and abiotic stresses in the amphibians (Fitzpatrick and Allison, 2014; Hernández-Gómez et al., 2020). In this study, we hypothesized that bacteria isolated from the skin of frogs may offer protective effect against the necrotrophic plant pathogen *Botrytis cinerea*.

To the best of our knowledge, only one study has reported the characterization of three bacteria isolated from Japanese frogs species as biocontrol agents against the fungus *C. orbiculare* (Susilawati et al., 2021). Similarly, in this work we analyzed the inhibitory effect of bacteria isolated from a tropical amphibian as biocontrol agents against *B. cinerea*, the causative agent of grey mold disease (Dean et al., 2012). Previously, we identified three different bacterial species that can inhibit the growth of *B. cinerea.* Our findings showed that compounds released by the bacteria modified the growth of the fungus at both early and late developmental stages. Numerous studies have demonstrated the potential of bacteria as biological control agents, including species of *Bacillus* spp., *Pseudomonas*, spp., and *Acinetobacter* spp., have been characterized for their effect in inhibiting *B. cinerea* (Trotel-Aziz et al., 2008; Kefi et al., 2015; Wang et al., 2020; Cevallos et al., 2022). *Bacillus* spp. it has been identified that their biocontrol activity is due to their secondary metabolism and the production of compounds, such as iturin, surfactin, and fengycin, bacteriocins, antimicrobial peptides, and lipopeptides, polyketides and siderophores, that participate in the inhibition of *B. cinerea* (Fira et al., 2018; Kiesewalter et al., 2021; Lu et al., 2021).

In addition to exerting antagonism mechanisms directly against pathogens, bacteria can also act indirectly through elicitors known as Microbial- or Pathogens-Associated Molecular Patterns [MAMPs or PAMPs], that are recognized through Pattern Recognition Receptors [PPRs] presents in the cell membrane plant, which activate the innate immunity (De Coninck et al., 2015; Wang et al., 2016). The MAMPs or PAMPs include the activation of hormonal pathways such as salicylic acid [SA], jasmonic acid [JA] and ethylene [ET], and consequently the induction of systemic defense responses (Robert-Seilaniantz et al., 2011). Studies have been conducted in recent years to demonstrate that the application of beneficial bacteria to plants activates their defense pathways, thereby protecting them against pathogenic fungi. Nie et al. (2017) demonstrated that the application of a *Bacillus cereus* AR156 strain to roots significantly reduced the incidence of *B. cinerea* disease by activating Induced Systemic Resistance [ISR]. Similarly, our results indicate that C23F, C26G, and C32I bacteria induce defense mechanisms in *A. thaliana* plants, leading to a decrease in *B. cinerea* disease. Since the bacteria was applied to the roots, while *B. cinerea* colonized the leaves, this suggests a possible activation of ISR, resulting in the observed resistance to infection by the pathogen.

Defense activity can trigger a series of events involving the transcriptional activation of hormone-related defense genes (AbuQamar et al., 2017; Lahlali et al., 2022). To determine changes in plant expression in response to the presence of C32I bacterium or/and *B. cinerea*, we analyzed the expression profiles between bipartite and tripartite interactions. Our analysis showed that DEGs are clustered into functional groups in response to biotic stimuli, jasmonic acid, and fungi (**Figure 6C**). During the plant-pathogen interaction, a decrease in the expression of genes related to the jasmonic acid pathway and the response to fungi was observed. Similarly, we observed an induction of genes related to pathogen recognition, which were triggered in the plant in response to *B. cinerea*. Previous studies report the induction of genes involved in the Induced Systemic Resistance [ISR], the jasmonic acid [JA], salicylic acid [SA], and ethylene [ET] hormone biosynthesis pathways, as well as other defense genes at 24 hpi (AbuQamar et al., 2006; Mulema and Denby, 2012; Sham et al., 2014; Xiong et al., 2018). In general, the authors report that gene expression initiates during the first hours of infection; however, a higher increase in the expression of these genes is observed at 24 hpi, which could explain under regulation of genes at 6 hpi in our study. Therefore, our results suggest a specific role of defense-related genes during the *A. thaliana - B. cinerea* interaction in the first hours of interaction.

In the case of the plant-bacteria interaction [*Acinetobacter* sp. *C32I*], we observed an increase in the number of up-regulated DEGs, which participate in the biosynthesis of growth hormones such as auxins [AUX], brassinosteroids [BR], and cytokinins [CK] [Romero et al., 2023, under review]. Additionally, we found that the bacteria can induce the expression of genes related to jasmonic acid, salicylic acid, and ethylene (**Figure 7**). Previous studies have reported the involvement of beneficial bacteria in the induction of the plant immune system, potentially facilitating the colonization processes of beneficial bacteria and enhancing defense against pathogens (Tzipilevich et al., 2021; Chakraborty, 2023). When analyzing the genes expressed during tripartite interactions, we observed an increased expression of genes related to defense hormones, response to pathogens, and other genes associated with oxidative stress [UPOX and ZAT12], which could be involved in the induction of defense through ISR (Davletova et al., 2005; Vaahtera et al., 2014). However, the increase in the expression observed in the plant-bacteria interaction, suggested that it could function as an ISR-priming, resulting in an accelerated activation of the defense response (Van der Ent et al., 2009; Zamioudis and Pieterse, 2012). The same effect has been observed with other biocontrols, where the inoculation of the fungus *Trichoderma hamatum T382* in *A. thaliana* induces early resistance against *B. cinerea*, which is subsequently moderated in response to the pathogen (Mathys et al., 2012). Similarly, was observed in the treatment of *A. thaliana* with the *Pseudomonas fluorescens bacterium PTA-CT2*, which induced the pre-infection plant defense against the pathogen *Pseudomonas syringae Pst DC3000* (Nguyen et al., 2020). Interestingly, a comparison of transcriptomic data from our results with other biocontrol agents, such as *Bacillus valezensis FBZ42, Pseudomonas fluorescences SS01*, and *Burkholderia* sp. *SSG*, shows transcriptomic differentiation, indicating that each strain elicits a plant-specific response and may be involved in ISR activation (van de Mortel et al., 2012; Tzipilevich et al., 2021; Kong et al., 2022). Based on this evidence, we can infer that the early activation of the plant defense system could contribute to an effective inhibition of *B. cinerea*.

It has been shown that ISR can be mediated by non-pathogenic bacteria that contribute to counteract the effects of pathogens, and this process is regulated by defense hormones (Pieterse et al., 1998; Van der Ent et al., 2009; van de Mortel et al., 2012). Interestingly, we observed using *A. thaliana* mutants that defense against *B. cinerea* is JA/ET-dependent and not SA-dependent. These results agree with previous reports suggesting that mutants with blocked ethylene [*ein2*] and jasmonic [*jar1*] signaling show increased susceptibility to *B. cinerea*. On the other hand, in the salicylic acid mutant [*eds5-1*], no significant impact on lesion size was observed (Ferrari et al., 2003). In other systems, it has been observed that JA [*jar1*] and ET [*ein2*] mutant plants treated with *Penicillium* sp. *GP16-2* did not reduce symptoms caused by *Pseudomonas syringae pv. tomato DC3000* (Hossain et al., 2008). Although there is no clear correlation between the expression levels of any of the genes and the degree of susceptibility to *B. cinerea*, the use of mutants inoculated with the C32I can provide information on the mechanisms used by the plant to defend itself against *B. cinerea* attack.

Studies have shown that the application of bacteria on agronomically important crops can serve as a biocontrol agent against *B. cinerea* to prolong fruit life during postharvest (Donmez et al., 2011; Lu et al., 2021; Chacón et al., 2022). To test the suppression of gray mold disease in fruits during postharvest and serve as inducers to elicit defense responses, we found that cells and bacterial filtrates of C23F, C26G, and C32I protect blueberry fruits against *B. cinerea* infection. Susilawati et al. (2021) showed HjD57, HjD92 and B341 from Japanese frogs-skin could reduce disease of the pathogen *Colletotrichum orbiculare* on cucumber plants. Similarly, tomato leaf assays that were inoculated with frog skin bacteria showed increased resistance to the pathogen. These results correlate with previous studies where the application of beneficial bacteria has been observed to reduce leaf disease severity (Kefi et al., 2015; Toral et al., 2020). Overall, the latter assay showed inhibitory activity of frog skin bacteria in the control of gray mold on plants of agronomic interest, therefore, an effective and feasible method to prevent postharvest fruit diseases is proposed.

## Conclusion

Bacteria as biocontrol agents present antagonistic mechanisms that inhibit the development of pathogens. However, little has been explored on bacteria from animals as potential fungicides. In this work, we demonstrate the inhibitory effects of three bacteria isolated from amphibian skin identified as *Acinetobacter* sp. C26G and C32I, and one *Enterobacteraceae* C23F on the growth and development of the plant pathogen *B. cinerea*. Additionally, we observed a reduction in the degree of infection upon the application of bacterial cells and filtrates. Moreover, the inoculation of the C32I bacteria to *A. thaliana* plants induced defense mechanisms by activating various defense genes associated with hormones such as salicylic acid [SA], jasmonic acid [JA], and ethylene [ET]. These activated defense pathways effectively protected the plant against *B. cinerea*. Based on these findings, we propose these bacterial strains as potential candidates for biological control agents, presenting an efficient and feasible method to prevent fruit diseases by inhibiting pathogen development and contributing to plant defense through immune system induction.

## Data availability statement

The datasets presented in this study can be found in online repositories. The names of the repository/repositories and accession number(s) can be found in the article/**Supplementary Material**.

## Author contributions

YR-C: Conceptualization, Formal analysis, Investigation, Validation, Writing – original draft, Writing – review & editing. FG: Data curation, Formal analysis, Writing – review & editing. DF: Conceptualization, Data curation, Formal analysis, Investigation, Writing – review & editing. WA: Data curation, Formal analysis, Writing – review & editing. FC: Conceptualization, Investigation, Methodology, Writing – review & editing. MT: Investigation, Methodology, Writing – review & editing. MC: Conceptualization, Supervision, Writing – review & editing. JD: Conceptualization, Formal analysis, Methodology, Supervision, Writing – review & editing. EAR: Conceptualization, Formal analysis, Funding acquisition, Investigation, Resources, Writing – original draft, Writing – review & editing. MS: Conceptualization, Funding acquisition, Investigation, Project administration, Supervision, Writing – original draft, Writing – review & editing.

## Supplementary material

Supplementary Figure 1 Biocontrol activity of frog skin bacteria against *B. cinerea* by the detached leaf test. Five-week-old tomato leaves treated with each bacterium were collected were infected with spore suspension of *B. cinerea*, after three days of incubation the lesion was evaluated. **(A)** Representative images of each treatment are shown. **(B)** Infection was assessed by measuring the lesion area. The graphs represent three biological replicates [n=30 ± SD]. Letters indicate a statistically significant difference, according to a one-way analysis of variance [ANOVA] [*p* ≤ 0.05] followed the Tukey test. Mock represents the plants with MS medium. Scale bar 1 cm.

Supplementary Data Sheet 1 List of differentially expressed genes for each condition, associated to .

Supplementary Data Sheet 2 List of differentially expressed genes [DEGs] in the bipartite and tripartite interactions [up-regulated and down-regulated], associated to .

Supplementary Data Sheet 3 Gene Ontology [GO] term enrichment list of differentially expressed genes associated to .
