# Supplementary figures and images for: Amphibian skin bacteria display antifungal activity and induce plant defense mechanisms against Botrytis cinerea
Source: Front Plant Sci. 2024 Apr 9;15:1392637. doi: 10.3389/fpls.2024.1392637 (PMC11035788; doi:10.3389/fpls.2024.1392637)

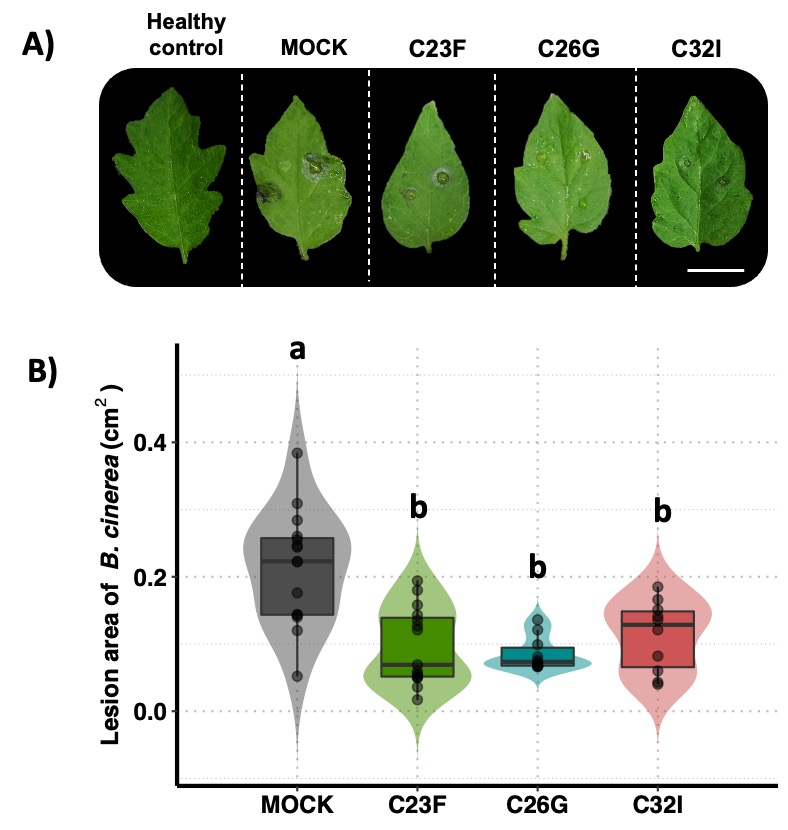

Supplement: Supplementary Figure 1 — Biocontrol activity of frog skin bacteria against B. cinerea by the detached leaf test. Five-week-old tomato leaves treated with each bacterium were collected were infected with spore suspension of B. cinerea, after three days of incubation the lesion was evaluated. (A) Representative images of each treatment are shown. (B) Infection was assessed by measuring the lesion area. The graphs represent three biological replicates [n=30 ± SD]. Letters indicate a statistically significant difference, according to a one-way analysis of variance [ANOVA] [p ≤ 0.05] followed the Tukey test. Mock represents the plants with MS medium. Scale bar 1 cm. [file Image_1.tiff]
